# Global output of research on the health of international migrant workers from 2000 to 2017

**DOI:** 10.1186/s12992-018-0419-9

**Published:** 2018-11-08

**Authors:** Waleed M. Sweileh

**Affiliations:** Department of Physiology, Pharmacology/Toxicology, Division of Biomedical Sciences, College of Medicine and Health Sciences, An-Najah National University, Nablus, Palestine

**Keywords:** Migrant workers, Health, Bibliometric analysis, Research activity

## Abstract

**Background:**

Approximately 150 million international migrant workers work under conditions that increase their risk of illness and injuries. The current study aimed to assess and analyze the global output of research on the health of international migrant workers to promote national and international policies that could help improve the health of international migrant workers.

**Methods:**

A bibliometric methodology was implemented using Scopus database after retrieving documents relevant to the health of migrant workers during the study period from 2000 to 2017.

**Results:**

In total, 955 documents were retrieved. The mean number of authors per document was 4.5 while the mean number of citation per document was 10.2. The retrieved documents were mainly in health policy and systems (*n* = 452; 47.3%), infectious diseases (*n* = 252; 26.4%), and mental and psychosocial health (*n* = 239; 25.0%). The health of Latino migrant farmworkers represented the largest cluster of keywords. The USA led (*n* = 389; 40.7%) with regard to the number of publications followed by China (*n* = 86; 9.0%) and the UK (*n* = 66; 6.9%). Researchers from the USA and Spain dominated the field. There were limited international research collaboration and a limited number and size of research networks. The *American Journal of Industrial Medicine* was most active (7.1%; *n* = 68) in publishing documents on health of migrant workers while the *Wake Forest University* was the most active (10.9%; *n* = 104) institution in this topic.

**Conclusion:**

The volume of global research output on the health of migrant workers was low. There was inadequate research on non-communicable diseases and maternal health of migrant workers. International research collaboration and the number of research networks were limited. Role of several world regions, particularly Arab region with 11% of international migrant workforce was also limited. There is an urgent need to prioritize research on migrant workers, especially female migrant workers in regions with low research contribution.

## Background

In the past decade, there has been a significant increase in research about international migration due to its enormous economic, social, cultural, and health impact on both sending and receiving countries [1]. The term international migrant refers to any person who is moving or has moved across international borders [2, 3]. The total number of international migrants has reached 258 million in 2017 [4, 5]. Of this large number of international migrants 106 million were born in Asia, 61 million were born in Europe, 38 million were born in Latin America and the Caribbean, and 36 million were born in Africa. At the country level, India was the largest country of origin of international migrants (17 million), followed by Mexico (13 million). Other countries of origin with large migrant populations include the Russian Federation (11 million), China (10 million), Bangladesh (7 million), Syrian Arab Republic (7 million), and Pakistan and Ukraine (6 million each).

International migrants comprise a wide range of populations, such as workers, refugees, students, undocumented migrants and others. Each type of migrants has different health needs and stressors. Health needs of this large number of people are important public health challenges faced by both sending and destination countries. Migration for work is the most common form of migration, particularly in a globalized world with profound economic and political disparities. Geographic proximity and historical links are also important motivating factors for the flow of migrant workers, particularly within the same region [3, 5].

The term migrant worker refers to all international migrants who are currently employed or are unemployed and seeking employment in their present country of residence [6]. According to the International Labour Organization [6], there are 150.3 million migrant workers in the world, with most living in high-income countries and many engaged in the service sector. Gender distribution showed that 55.7% of international migrant workers are males and 44.3% are females. Almost half of the migrant workers are concentrated in Northern America, Northern, Southern and Western Europe. The Arab region accounts for 11.7% of all migrant workers. Most international migrant workers are found in agricultural, construction, food processing, and other low-skilled jobs. It is estimated that 16.7 million international migrants were part of the agricultural labor workforce worldwide [6].

International migrant workers contribute positively to the economic situation of source countries through sending remittances [7, 8]. Furthermore, migrant workers develop skills, learn new technologies, and make new social and professional connections that could benefit their home countries upon their return [9]. However, the health of migrant workers and the families they leave behind have not been given enough consideration by health-system planners in both sending and destination countries. Migrant workers are most vulnerable to health risks because they are often engaged in what is known as 3-D jobs—dirty, dangerous, and demanding (sometimes degrading or demeaning) [6]. For example, international migrant workers face several occupational hazards due to lack of training and/or appropriate safety measures [10]. Female migrant workers may be subjected to various types of violence and sexual exploitation [11, 12]. Furthermore, migrant workers might face cultural and language barriers which could negatively affect the quality of healthcare services they receive [13–17]. A new dimension in the field of migrant workers health is the exploitation and abuse of labor and sex trafficked men, women and children, with negative health consequences on individuals and national health security [18–20]. In developed countries, such as the USA and UAE, there are employer agencies that recruit international workers from low- and middle-income countries (LMIC) by making fraudulent and false promises. Those migrant workers might end up being abused, slaved, mistreated, and deprived of basic medical and healthcare services. It is common for female migrant workers in some destination countries to end up as victims to sexual violence and harassment [21].

Despite the large numbers of migrant workers and the serious health threats they are facing, there has not been any assessment of the peer-reviewed literature on the health of migrant workers. Unfortunately, certain governments have the misconception that research about migrant workers could negate developmental gains or damage their political and human right reputation [22]. Furthermore, a recent study on the global migration health called for research assessment of migration health of all categories of international migrants to spot gaps for intervention and future policy implementation [23]. Therefore, the aim of this study was to assess and analyze the global output of research on the health of international migrant workers to spot research gaps, enrich the international research agendas pertaining to migration health, and create a research repository on this topic. Specifically, the study will examine the growth of publications, authorship analysis, geographical distribution, international research collaboration, important themes discussed, and highly cited articles. The overall aim of this study is to provide baseline data on health-related research issues on international migrant workers for future planning and comparison. Furthermore, this study is in agreement with the 2030 global agenda of health and human rights set in Sustainable Development Goals (SDGs) [24].

## Methods

### Database

A bibliometric methodology was implemented in this study. SciVerse Scopus was used to accomplish the objective of the current study since it was previously used in several bibliometric studies including those pertaining to migration health [25–33]. As a database, Scopus is larger than Web of Science and is 100% inclusive of Pubmed. Furthermore, Scopus has several functions that facilitate research trends, citation analysis, mapping of keywords, and international research collaboration.

### Study duration and language

The duration of the study was set from January 2000 until December 2017. This was judged to capture most studies on the health of migrant workers. Scopus allows researchers to filter the retrieved documents based on the language of the documents. Yet, in this study, no language restriction was made and all retrieved documents, regardless of their language, were analyzed. Documents appearing in Scopus must have an English abstract, and therefore, the relevancy of any retrieved document could be confirmed by reading the English abstract regardless of the original language of the document.

### Search strategy

The search strategy utilized several different keywords in title search. Examples of keywords used include “*migrant *worker” or “*migrant laborer” or “*migrant labourer*” or (“*migrant” and *worker*) or (*migrant and laborer*) or (“migratory worker*”). The author was careful to use all potential keywords related to migrant workers. Therefore, a survey of literature on migrant workers was carried out before deciding on keywords [34–40]. Furthermore, the keywords used in the current study were partially extracted from the bibliometric study by Sweielh et al. on global migration health in which migrant workers’ health was one component [23]. The health component of the search strategy was defined as documents classified by Scopus to be within the following subject areas: medicine, psychology, nursing, biochemistry, immunology, pharmacology, toxicology, dentistry, and neuroscience. These subject areas are available through Scopus and can selected to refine the retrieved documents to health component.

### Exclusion criteria

The current study focused on international migrant workers and therefore documents about internal migrants or documents pertaining to urban-to-rural or rural-to-urban documents were excluded. Furthermore, documents pertaining to health professionals such as nurses or physicians (brain drain) were also excluded because this category is usually well-paid and has better working conditions than the bulk of international migrant workers.

### Validity check

The validity of the search strategy was explained in previously published studies [23, 41]. The validity check consisted of two approaches, one to confirm the absence of false positive results and one to confirm the absence of false negative results. To confirm absence of false positive results a sample of top 100 cited documents were reviewed. For the absence of false negative results, the number of documents for each of the top ten active authors was compared with the number present in the personal profile of each of the top ten active authors. A correlation coefficient greater than 0.95 is indicative of the absence of false positive results.

Once the retrieved documents were refined and all false positive documents were excluded, a set of analytic functions were implemented to produce the required bibliometric indicators.

### Research domains

For the classification of research domains of the retrieved documents, five domains were created: health policy and systems, mental and psychosocial health, maternal and reproductive health, non-communicable diseases, and infectious diseases. These domains fall within the large domain of public health. However, this classification better serves the analysis for finding research gaps. The health policy and systems domain included documents in health system, health services, access to healthcare, human rights, rights to health, hospitalization, medical services, emergency room visits, occupational health, and migration policies [1]. Occupational health documents were categorized under the domain “health policy and systems” because it is a consequence of job regulations and availability of health insurance and health services.

### Bibliometric analysis and indicators

Retrieved data were exported into Microsoft Excel for calculation and tabulation. Scopus has several functions that can generate bibliometric indicators. Such functions include annual growth, citations, source title, country list, author list, institution list, and types of documents. For data visualization, the retrieved documents were visualized using VOSviewer (version 1.6.8; Leiden University, the Netherlands) to create three types of maps: keyword occurrences, international research collaboration, and author research network [42]. ArcMap 10.1 (Esri, California, USA) is a GIS program that was used to map the distribution of retrieved documents based on author affiliation.

All through the manuscript, frequency and percentages were provided in tables. For line of best fit and best line equation, Statistical Package for Social Sciences (IBM SPSS statistics; version 21; Armonk, N.Y: IBM Corporation) program was used. For the impact of the retrieved documents, Hirsh index (*h*-index) was used [43]. The rank of each journal in the top active list was obtained from Scimago Journal Rank website [44]. The impact factor (IF) of the most active publishing journal was obtained from the most recent Journal Citation Report.

### Research ethics

Finally, the current study was based on electronic data and no human subjects were directly involved and therefore the ANU-IRB office did not require an approval of the study.

## Results

### Types of documents

Nine hundred and fifty-five documents were retrieved based on the methodology described. Research articles (85.5%; *n* = 817) constituted the majority of retrieved documents followed by review articles (6.0%; *n* = 57), and letters to the editor (2.5%; *n* = 24) (Table 1).

**Table 1:** Types of retrieved documents on health of migrant workers (2000–2017)

Type of document	Number of documents *N* = 955	%
Article	817	85.5
Review	57	6.0
Letter	24	2.5
Note	21	2.2
Conference Paper	13	1.4
Editorial	10	1.0
Short Survey	6	0.7
Article in Press (unidentified type)	7	0.6

### Citation analysis and top cited documents

The retrieved documents received 9725 citations, an average of 10.2 citations per documents. The *h*-index of the retrieved documents was 42. The top 10 cited documents were either in the mental and psychosocial health [45–48] or in the health policies and systems, specifically in occupational health [49–53] (Table 2). The document that received the highest citations (150 citations) was published in the *American Journal of Public Health* [48]. The document discussed the prevalence of psychiatric disorders among Mexican migrant farmworkers in California. The top 10 documents included one document in infectious diseases [54].

**Table 2:** Top 10 cited documents on health of migrant workers (2000–2017)

SCR^a^	Title	Year	Source	Number of citations
1st	Lifetime prevelance of and risk factors for psychiatric disorders among Mexican migrant farmworkers in California	2000	American Journal of Public Health	150
2nd	Psychosocial stressors associated with Mexican migrant farmworkers in the midwest United States.	2003	Journal of immigrant health	121
3rd	Immigrant populations, work and health - A systematic literature review	2007	Scandinavian Journal of Work, Environment and Health	116
4th	Work characteristics and pesticide exposures among migrant agricultural families: A community-based research approach	2001	Environmental Health Perspectives	108
4th	Agricultural injury in California migrant Hispanic farm workers	2003	American Journal of Industrial Medicine	108
6th	Occupational risks and injuries in non-agricultural immigrant Latino workers	2002	American Journal of Industrial Medicine	103
7th	Use of commercial sex workers among Hispanic migrants in North Carolina: Implications for the spread of HIV	2004	Perspectives on Sexual and Reproductive Health	101
8th	Psychosocial predictors of anxiety among immigrant Mexican migrant farmworkers: Implications for prevention and treatment	2002	Cultural Diversity and Ethnic Minority Psychology	91
9th	Leaving family for work: Ambivalence and mental health among Mexican migrant farmworker men	2006	Journal of Immigrant and Minority Health	89
10th	Farmworker reports of pesticide safety and sanitation in the work environment	2001	American Journal of Industrial Medicine	76

*SCR* Standard competition ranking;

^a^Equal citations have the same ranking number, and then a gap is left in the ranking numbers

### Research domains

Analysis of the retrieved literature revealed that 239 (25.0%) documents were on mental and psychosocial health of migrant workers; 452 (47.3%) documents on health policy and systems (including occupational health), 24 (2.5%) documents on non-communicable diseases (NCD); 252 (26.4%) on infectious diseases; 111 (11.6%) on maternal and reproductive health (Table 3). There is an overlap among various research domains, which made the total percentage to be more than 100%.

**Table 3:** Research domains of the retrieved documents (2000–2017)

Research domain	Number of publications (%) *N* = 955
Mental and psychosocial health	239 (25.0%)
Health policy and systems^a^	452 (47.3%)
Non-communicable diseases (NCD)	24 (2.5%)
Infectious diseases	252 (26.4%)
Maternal and reproductive health	111 (11.6%)

^a^This domain includes occupational health-related documents

### Most frequent author keywords

**Fig. 1:**
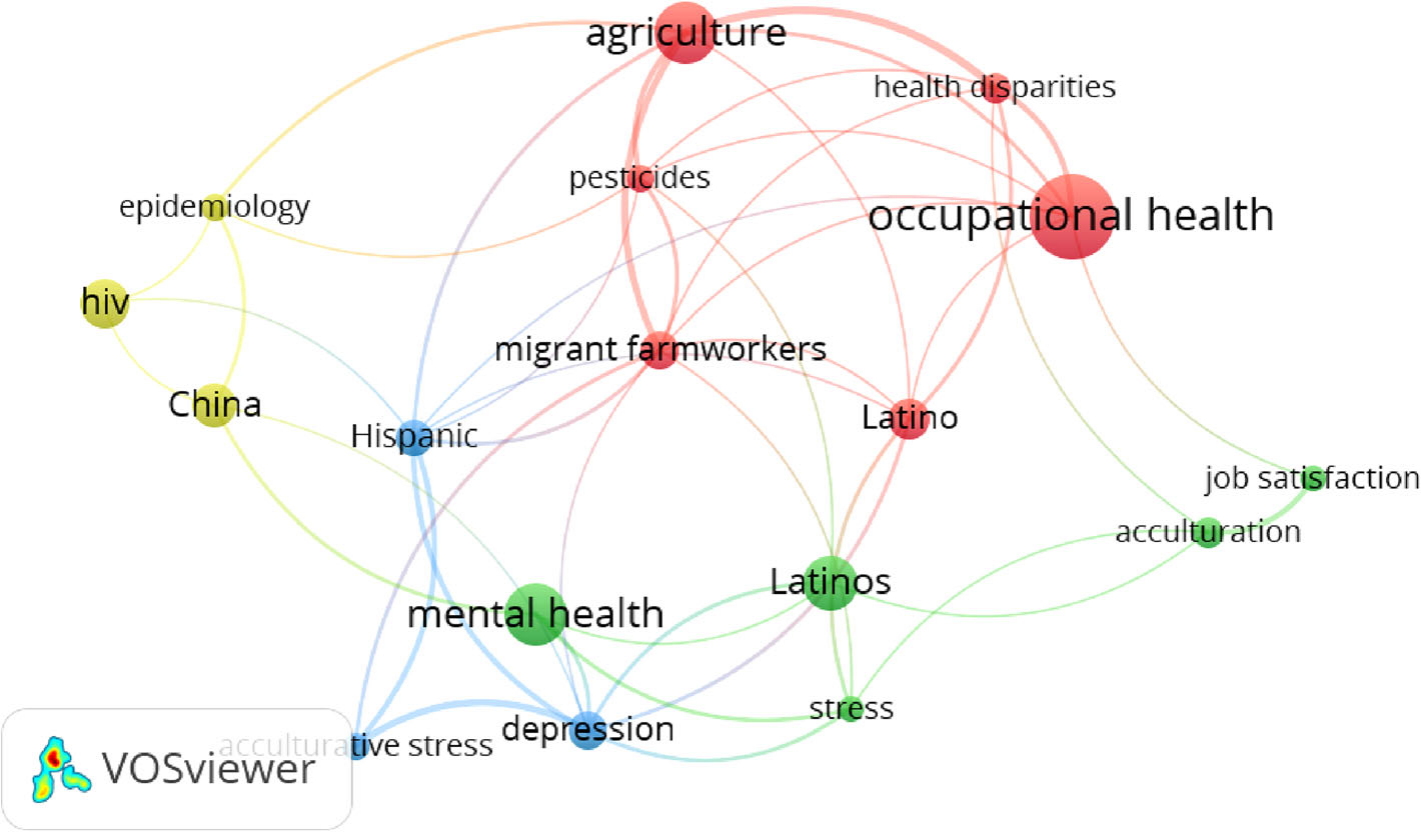
Visualization map of keywords with minimum occurrence of 10

Mapping keywords with minimum occurrences of 10 yielded a map with 17 items distributed in four clusters (Fig. 1). The largest cluster focused on the occupational health of Latino migrant farmworkers. The blue and green clusters focused on the mental and psychosocial health of Latino/Hispanic migrant workers. The fourth cluster focused on the epidemiology of HIV in China in relation to migrant workers.

### Growth of publications

**Fig. 2:**
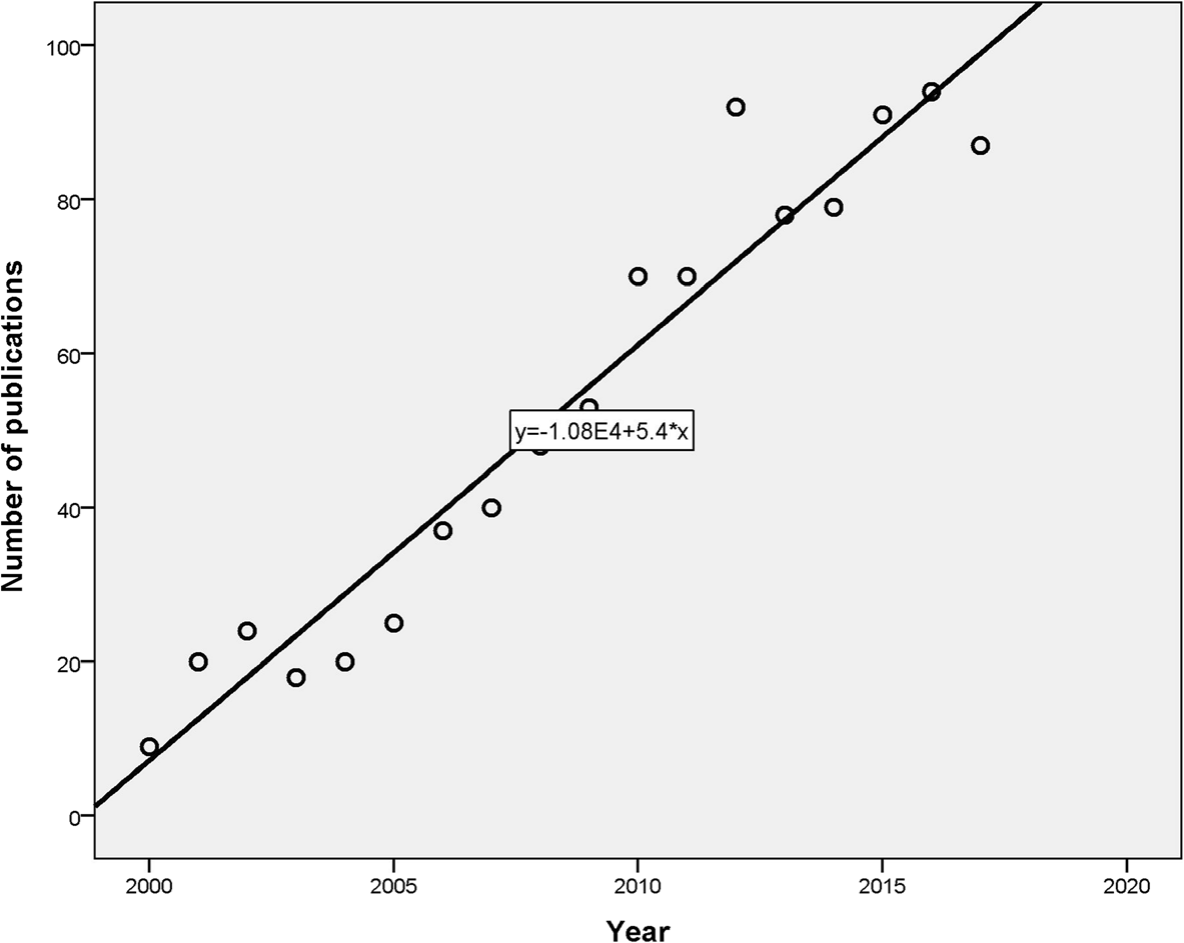
Lines of best fit for annual growth of publications

The number of publications showed slow increments over time. Plotting the number of publications versus time yielded a straight line with R^2^ = 0.95 (Fig. 2). According to the equation of the straight line (Y = 5.4X – 10,800), it is expected that the number of publications in 2020 and 2030 on migrant workers’ health to be approximately 108 and 162 respectively; i.e. an increase by 50% during a decade interval (2020 to 2030).

### Top 10 active countries

**Fig. 3:**
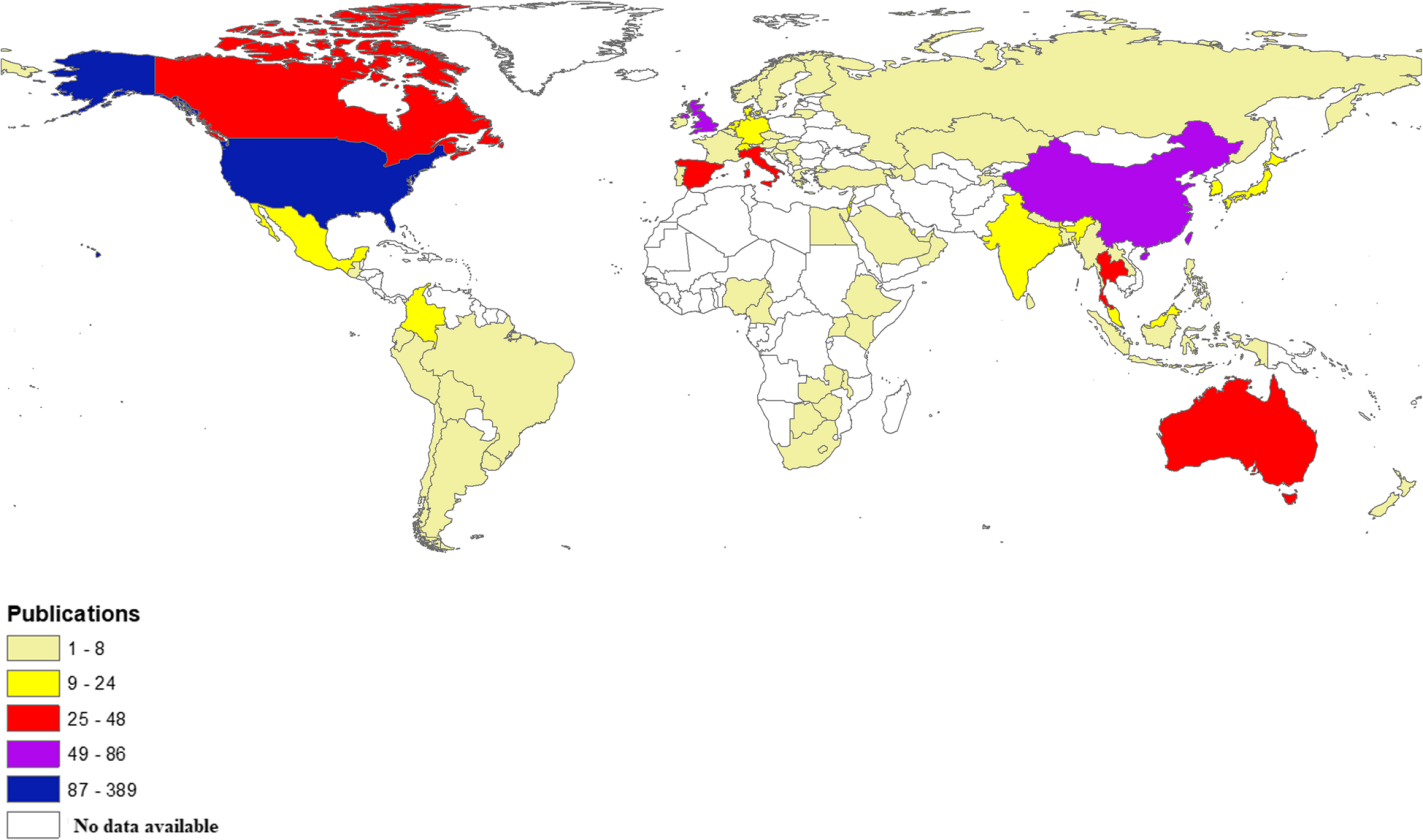
Geographic distribution of publications on health of migrant workers. The color coding is as follows

Authors from 77 different countries participated in publishing the retrieved documents. The contribution of US researchers was the highest (389; 40.7%) followed by those from China (86; 9.0%) and the UK (66; 6.9%). The list of top 10 active countries (Table 4) included three countries in South Eastern Pacific region, two countries in South East Asia, three countries in Western Europe, and two countries in Northern America. None of the active countries was from South America or Eastern Mediterranean, or Eastern Europe, or Africa. Geographic distribution of publications based on the country affiliation of all authors showed the prominent contribution of the US, Canada, Australia, certain European countries, and certain countries in South-Eastern Asia (Fig. 3).

**Table 4:** Top 10 active countries on migrant workers’ health research (1988–2017)

SCR	Country	Number of publications (%); *N* = 955
1st	United States	389 (40.7)
2nd	China	86 (9.0)
3rd	United Kingdom	66 (6.9)
4th	Canada	48 (5.0)
5th	Thailand	46 (4.8)
6th	Spain	40 (4.2)
7th	Australia	34 (3.6)
8th	Italy	33 (3.5)
9th	Malaysia	24 (2.5)
10th	India	23 (2.4)

*SCR* Standard competition ranking

### International research collaboration

**Fig. 4:**
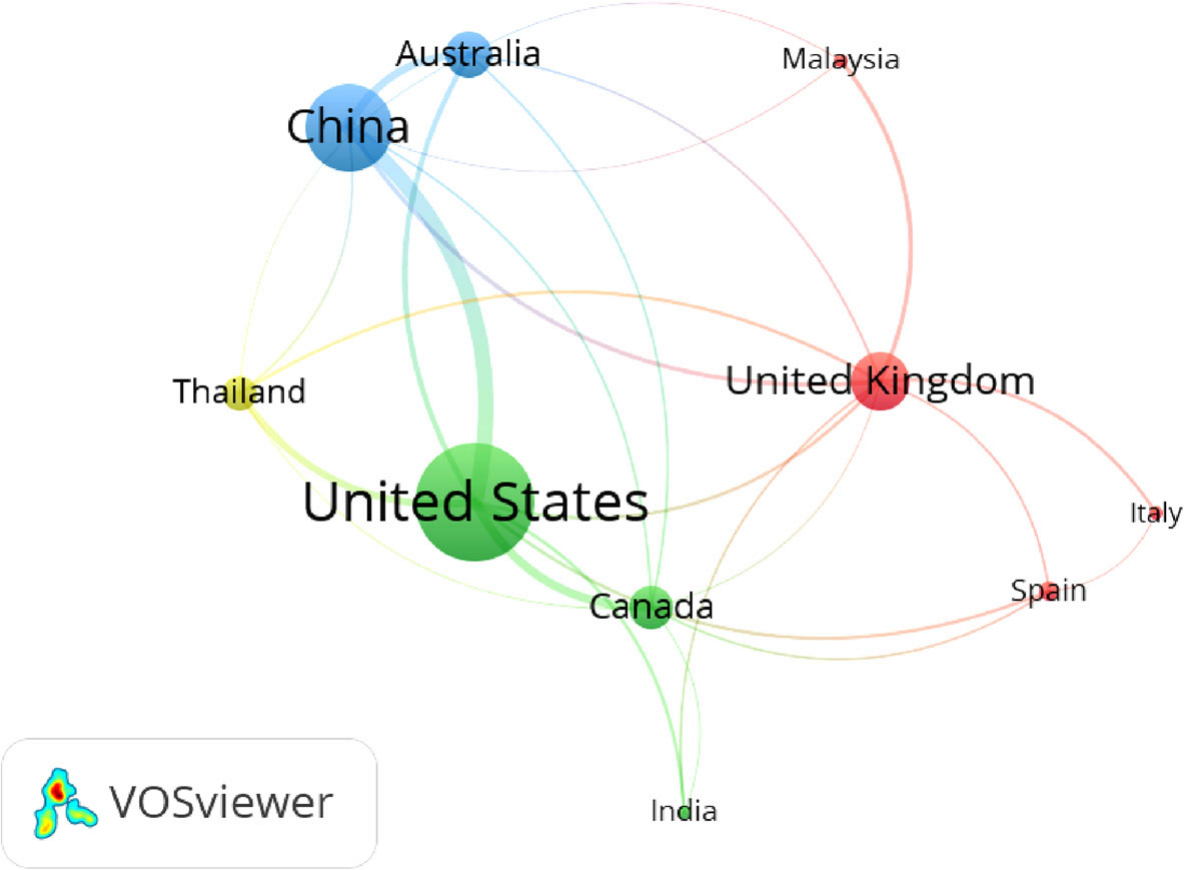
International research collaboration among top 10 active countries. Thickness of the connecting line represents strength of research collaboration. Similar color represents research relatedness

The top 10 active countries showed limited research collaboration as visualized by the thickness of the connecting lines between the countries. Relatively speaking, research collaboration in migrant workers’ health research was the strongest between the USA and China (Fig. 4).

### Top 10 active authors and research networks

**Fig. 5:**
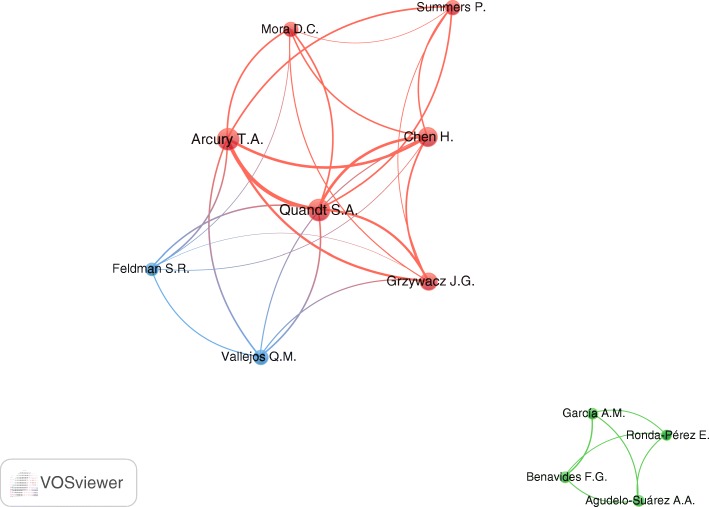
Research networks among top 10 active authors

In total, 4300 authors participated in publishing the retrieved documents, an average of 4.5 authors per article. Approximately 15% (*n* = 140) documents were single-authored publications while the remaining were multiple-authored publications. The top 10 active authors were listed in Table 5. Professors Arcury, T.A and Quandt, S.A (Wake Forest University School of Medicine, U.S) had a prominent role in health research of migrant workers. The top 10 active authors were mainly from the USA and Spain. Mapping active authors showed that they existed in two research networks (Fig. 5). The largest research network included eight authors; all were from the USA. The smaller research network included four researchers, mainly from Spain. Researchers in the large network were mainly from the same institution (Wake Forest University). Due to research collaboration within each research network, there is a lot of overlap in the number of publications produced by each researcher.

**Table 5:** Top 10 active countries on migrant workers’ health research (1988–2017)

SCR^a^	Author	Number of publications (%); *N* = 955	Affiliation as it appeared in Scopus profile
1st	Arcury, T.A.	101 (10.6)	Wake Forest University School of Medicine, Department of Family & Community Medicine, Winston Salem, U.S
2nd	Quandt, S.A.	96 (10.1)	Wake Forest University School of Medicine, Center for Worker Health, Winston Salem, U.S
3rd	Chen, H.	46 (4.8)	Wake Forest University School of Medicine, Center for Worker Health, Winston Salem, U.S
4th	Grzywacz, J.G.	40 (4.2)	Florida State University, Department of Family and Child Sciences, Tallahassee, U.S
5th	Benavides, F.G.	21 (2.2)	Universitat Pompeu Fabra, Centro de Investigación en Salud Laboral, Barcelona, Spain
6th	Summers, P.	19 (2.0)	Wake Forest University School of Medicine, Clinical and Translational Science Institute, Winston Salem, U.S
7th	Mora, D.C.	18 (1.9)	Wake Forest University School of Medicine, Department of Family & Community Medicine, Winston Salem, U.S
8th	Vallejos, Q.M.	17 (1.8)	The University of North Carolina at Greensboro, Department of Public Health Education, Greensboro, U.S
9th	Ronda-Pérez, E.	16 (1.7)	Universitat d’Alacant, Public Health Research Group, Alicante, Spain
10th	Agudelo-Suárez, A.A.	15 (1.6)	Universidad de Antioquia, Faculty of Dentistry, Medellin, Colombia
10th	García, A.M.	15 (1.6)	Universitat de Valencia, Valencia, Spain
10th	Feldman, S.R	15 (1.6)	Wake Forest University School of Medicine, Winston

*SCR* Standard competition ranking;

^a^Equal authors have the same ranking number, and then a gap is left in the ranking numbers

### Preferred journals for publishing documents on migrant workers’ health

The *American Journal of Industrial Medicine* was the most active (7.1%; *n* = 68). The list of active journals included seven journals in in public health, two in general medicine, and one in general science (Table 6). Half the active journals were published from the USA, four from Europe, and one from China. Four journals in the active list were categorized as high rank (Q1) journals. Eight journals in the active list were indexed in ISI-Web of Science and had an impact factor [55] according to Journal Citation Report (JCR; 2017). All journals in the active list were indexed in Scopus.

**Table 6:** Top 10 active journals in publishing documents on migrant workers’ health (2000–2017)

SCR^a^	Journal (Country)	Number of publications (%); *N* = 955	Subject area (category)	Rank^b^	Country	IF^c^
1st	American Journal Of Industrial Medicine	68 (7.1)	Medicine (Pubic health)	Q1	USA	1.729
2nd	Journal Of Immigrant And Minority Health	50 (5.2)	Medicine (Epidemiology/Pubic health)	Q3/Q2	Germany	1.284
3rd	Journal Of Agromedicine	29 (3.0)	Medicine (Pubic health)	Q2	USA	1.322
4th	BMC Public Health	20 (2.1)	Medicine (Pubic health)	Q1	UK	2.265
5th	Journal Of Occupational And Environmental Medicine	15 (1.6)	Medicine (Pubic health)	Q2	USA	1.355
5th	Plos One	15 (1.6)	Agricultural and Biological Sciences (Miscellaneous)	Q1	USA	3.54
7th	Chinese Journal Of Industrial Hygiene And Occupational Diseases	13 (1.4)	Medicine (Miscellaneous)	Q4	China	NA
8th	AIDS And Behavior	12 (1.3)	Medicine (Infectious disease/Public Health/ Social Psychology)	Q1/Q1	Netherlands	3.017
8th	New Solutions A Journal Of Environmental And Occupational Health Policy Ns	12 (1.3)	Medicine (Miscellaneous)	Q3	USA	NA
10th	International Journal Of International Journal of Environmental Research And Public Health	11 (1.2)	Medicine (Toxicology/Public Health)	Q2/Q2	Switzerland	2.145

*SCR* Standard competition ranking, *IF* Impact Factor, *NA* Not available

^a^Equal journals have the same ranking number, and then a gap is left in the ranking numbers

^b^Journal rank was obtained from Scimago Journal rank. Q1 = highest rank; Q4 = lowes rank

^c^The impact factor was reported according to journal citation reports (JCR) 2017

### Top 10 active institutions

The top 10 active institutions were led by *Wake Forest University* (10.9%; *n* = 104) followed by two universities in Spain (Table 7). The active list included six institutions in the USA (four academic and two non-academic institutions). The list also included three academic institutions in Spain, one in Thailand, one in China, and one in Colombia. Two of the active institution (*Wake Forest University* and The *University of North Carolina at Greensboro*) were based in North Carolina (USA).

**Table 7:** Top 10 active institutions in migrant workers’ health research (2000–2017)

SCR^a^	Institution	Number of publications (%); *N* = 955	Country
1st	Wake Forest University	104 (10.9)	USA
2nd	Universitat d’Alacant	22 (2.3)	Spain
3rd	Universitat Pompeu Fabra	21 (2.2)	Spain
4th	Centers for Disease Control and Prevention	20 (2.1)	USA
5th	University of Washington, Seattle	19 (2.0)	USA
6th	Sun Yat-Sen University	16 (1.7)	China
7th	Mahidol University	15 (1.6)	Thailand
8th	Universitat de ValEncia	14 (1.5)	Spain
8th	National Institute for Occupational Safety and Health	14 (1.5)	USA
8th	The University of North Carolina at Greensboro	14 (1.5)	USA
8th	University of California, Berkeley	14 (1.5)	USA
8th	Universidad de Antioquia	14 (1.5)	Colombia

*SCR* Standard competition ranking;

^a^Equal institutions have the same ranking number, and then a gap is left in the ranking numbers

## Discussion

The current study aimed to analyze and assess published literature on the health of migrant workers. Such analysis helps in addressing and optimizing clear strategies in both sending and destination countries to protect migrant workers. The current study showed that the volume of the retrieved literature was relatively low. A bibliometric analysis of literature on global migration health conducted by Sweileh et al. showed that a volume of more than 20,000 documents was published on global migration health from 2000 to 2016 [23]. This means that literature on the health of migrant workers represents less than 5% of the overall literature. The relatively lower volume of literature on health of migrant workers could be due to the limited number of worldwide scholars interested in the health of migrant workers. Furthermore, the health issues of migrant workers are usually hidden ones and might be difficult to research given the idea that migrant workers tend not to talk about their negative experiences to avoid losing their jobs [56]. A third potential reason is the fact that serious health issues of massive numbers of refugees dominated the field of migration health [1].

Analysis of research domains of the retrieved literature showed that research about occupational health dominated the field. However, the number of publications could be much higher if research about migrant workers was not complicated by issues related to employees and hidden working conditions [56]. Migrant workers often serve in jobs that natives are reluctant to perform [57]. Thus, migrants are more often exposed to potentially health-damaging work environments than native workers [58]. A review study on the occupational health of migrant workers in the USA showed that the proportion of fatal and nonfatal workplace injuries has been increasing among migrant workers due to the shift in hazardous jobs to the immigrant workforce [59].

Publications in communicable diseases among migrant workers were also evident in the retrieved literature. One concern of health officials in destination countries is that the sending countries tend to have higher prevalence of infectious diseases and less screening systems, which increases the risk of disease importation [60]. The focus on infectious diseases among migrant workers is also based on the idea that migrant workers might have higher risk of infections than non-migrants probably due to risky sexual behaviors, socio-economic factors, their living conditions and financial, language and cultural barriers to healthcare access [61–63]. Both sending and destination countries need to have an improved screening and preventive measures to limit potential spread of certain serious infections carried by travelers [64]. For example, the rapid spread of pandemic (H1N1) influenza in Mexico led to fears among health officials in North America of potential transmission of the diseases by Mexican migrant workers. To avoid negative opinion and to contain the spread of the infection, health officials in Mexico and North America immediately implemented screening of migrant workers both at departure and arrival points.

The retrieved literature also included relatively good volume of publications on mental health of migrant workers and their families. The cultural shock and acculturative stress in addition to barriers to access healthcare services increase the risk of depression, stress and risk of suicide among migrant workers [65–68]. Furthermore, work environment that might include physical abuse and harassment is a potential cause for negative mental health outcomes [37]. The presence of several documents about occupational health in the top ten cited documents emphasized the good share of research on occupational health in the retrieved documents.

The current study showed relatively lower research activity on NCDs on migrant workers. One potential reason for that is the fact that migrant workers are usually young and might not have a high prevalence of NCD compared to elderly people. However, migrant workers might get engaged in risky life style such as chronic tobacco smoking or alcohol abuse that could lead to NCD [69]. Furthermore, those with existing chronic illness might experience deterioration of their chronic disease due to interrupted healthcare services or lack of access to medications and medical follow up [70–72]. Another important factor when considering NCD among migrant workers is affordability to cover for medications when needed which ultimately affects the health of migrant workers [55]. Maternal and reproductive health research domain was also dwarfed on the expense of occupational health and mental health domains. Female migrant workers often face more barriers related to reproductive healthcare, access to health services, child care, family planning services, prenatal care, and unplanned pregnancies than do native-born women [73, 74].

The current study showed that countries, researchers, and institutions in high-income destination countries contributed to a large percentage of retrieved documents. In destination countries, migration has important public health consequences that can affect the national healthcare system. Research experts and research budgets in destination countries are key reasons for the prominent research role of these countries. The findings in the current study showed that China is one of the top ten active countries in research about health of migrant workers. China has become not only a source, but also a destination for migrants from all over the world. There are 60 million overseas Chinese around the world according to the Annual Report on Chinese International Migration [75]. Other Asian countries such as Malaysia, India, and Thailand were also among top ten active countries in producing research about health of migrant workers. This was unsurprising given that more than 100 million of international migrants came from Asia [3, 76].

Despite that the Arab region with approximately 11% of the global migrant workforce had limited and even negligible contribution to literature on health of migrant workers. However, there is frequent worldwide media coverage around the South Asian construction workers building the football stadiums for the 2022 football world cup in Qatar.

The contribution of source countries to literature on migrant workers was low due to the that most of the source countries are poor or undergoing internal civil war or regional conflicts, which limits research productivity from such countries. Effective international health planning that covers migrant workers requires a certain extent of international research collaboration among different countries. Such collaboration was absent which affects collaborative efforts of screening migrant workers for potential contagious diseases that might spread by travelers. Furthermore, collaboration between sending and destination countries is required to set plans to face mental health problems for migrants and families they leave behind. Furthermore, both sending and destination countries need to collaborate to increase awareness of migrant workers about their legal and human rights at working places. Of paramount importance is the efforts directed to increase awareness about potential labor trafficking and exploitation into sex industry or dangerous labor. Finally, sending countries need to collaborate with destination countries to attract back skilled migrant workers to ensure sustainable development in sending countries.

The current study, up to the author’s best knowledge, is the first bibliometric analysis of literature on the health of migrant workers. However, the current study has a few limitations. Bibliometric analysis only picks up published literature in academic journals. There are some reports in grey literature, which may be informative for the field, but were missed in this analysis. In the current study, the research strategy was designed to retrieve the maximum number of documents with minimum false positive and negative results. However, the presence of false positive and negative results is always a possibility. The use of Scopus might have created some bias towards destination countries because journals indexed in Scopus are mainly in English and produced by developed countries. Finally, data obtained might have some percentage of errors due to Scopus updating process. Therefore, a margin of error needs to be considered when interpreting top active authors, institutions, and countries. The overlap due to intra-country collaboration needs to be considered since more than one active author might share similar research output. All these limitations are typical of any bibliometric analysis and are not unique to the current study [26, 41, 77–80].

## Conclusion

The current study showed that research on migrant workers’ health need to be strengthened and encouraged given that the volume and growth rate were relatively inadequate. Attention to research gaps such as the focus on NCD and maternal and reproductive health is also needed. International research collaboration and research networks that include countries of origin and receiving countries were deficient. Building such collaboration and networks require recruitment of large numbers of scholars from different parts of the world to the subject of migration health in general and migrant workers’ health in particular.

## Abbreviation

NCD: Non-communicable diseases
